# Differences in the Oral Microbiome Between Patients With and Without Oral Squamous Cell Carcinoma

**DOI:** 10.1111/jop.70099

**Published:** 2025-12-05

**Authors:** Satoshi Fukase, Atsumu Kouketsu, Toru Tamahara, Tatsuru Saito, Akiko Ito, Yutaro Higashi, Tomonari Kajita, Tsuyoshi Kurobane, Masaaki Miyakoshi, Masahiro Iikubo, Ritsuko Shimizu, Tetsu Takahashi, Kensuke Yamauchi, Tsuyoshi Sugiura

**Affiliations:** ^1^ Division of Oral and Maxillofacial Oncology and Surgical Sciences, Department of Disease Management Dentistry Tohoku University Graduate School of Dentistry Sendai Japan; ^2^ Tohoku Medical Megabank Organization Tohoku University Sendai Japan; ^3^ Division of Dental Informatics and Radiology Tohoku University Graduate School of Dentistry Sendai Japan; ^4^ Division of Oral and Maxillofacial Reconstructive Surgery, Department of Disease Management Dentistry Tohoku University Graduate School of Dentistry Sendai Japan

**Keywords:** metagenomics, microbiota, mouth neoplasms, saliva

## Abstract

**Background:**

Although studies have demonstrated a relationship between pathogenic microorganisms and oral cancer, no study has demonstrated a relationship between changes in bacterial flora and oral squamous cell carcinoma (OSCC). Therefore, we investigated the association between oral microbiota and oral squamous cell carcinoma using metagenomic analysis.

**Methods:**

Saliva samples from 64 patients with OSCC and 50 healthy controls who visited the Department of Oral Surgery, Tohoku University Hospital, were collected, and bacterial genomic DNA was extracted using polymerase chain reaction amplification. Single‐end sequencing was performed using the Illumina MiSeq platform, and sequence data were analyzed using the Quantitative Insights Into Microbial Ecology 2 platform. The Steel–Dwass test was used for between‐group comparisons, and Analysis of Compositions of Microbiomes with Bias Correction was used to detect significant differences in microbiome composition.

**Results:**

Significant differences were observed in alpha‐diversity indices of bacterial flora (richness, Faith‐ phylogenetic diversity, Shannon index) in the OSCC group compared to those in the control group. Among the OSCC group, patients with larger tumor diameters and lymph node metastases (T3/T4, N1 or greater) formed independent clusters in the beta diversity analysis of the bacterial flora. Bacteria of the *Actinomycetia* phylum, such as *Actinomyces* and *Rothia*, were significantly reduced in patients with higher stage and pathological grade. Conversely, bacteria of the phylum *Spirochaetia* and *Proteobacteria*, particularly those of the genus *Treponema*, were significantly elevated in advanced cancer cases.

**Conclusions:**

Our results suggest that changes in the oral microbiota may play a role in OSCC development and progression.

## 1 Introduction

The microbiome is one of the most important factors that contribute to the regulation of host health, and numerous studies have revealed the mechanisms by which microorganisms affect the host's general condition [1, 2, 3, 4, 5]. The intestinal microbiota is associated with several systemic diseases, including cancer, irritable bowel syndrome, cranial nerve diseases, diabetes, and ischemic cardiovascular diseases; however, no apparent relationship with bacteria has been identified in these diseases. In general, “microbiota” refers to the community of living microorganisms, whereas “microbiome” includes the microorganisms and their collective genetic material and functions. The oral microflora is the second most diverse after the intestinal microflora. Consequently, studies in dentistry have focused on the relationship among oral bacteria, dental caries, and periodontal disease; however, it is now evident that oral bacteria can also cause systemic diseases [4, 6, 7, 8]. The oral cavity is constantly exposed to microorganisms via saliva and is inhabited by more than 500 bacterial species [9]. While the diversity of bacterial species is lowest in the mucosal flora, followed by the saliva, it is highest in dental plaques.

Next‐generation sequencing and bioinformatics have made remarkable progress in information analysis technology [10]. Whole‐genome analysis of the oral microflora (bacterial flora), an unexplored field owing to the large number of bacterial species, has also experienced dramatic progress [11]. Additionally, recent advances in metagenomic sequencing and bioinformatics have significantly expanded our understanding of the role of the microbiome in systemic diseases, including cancer [12]. Notably, studies such as Han et al. [13] and Wang et al. [14] have demonstrated that microbial dysbiosis is closely associated with disease severity and progression in head and neck squamous cell carcinoma.

Moreover, recent studies on the relationship between solid tumors and bacterial flora provide clarity on the involvement of specific microorganisms in gastrointestinal tract carcinogenesis. For instance, in addition to the presence of *Helicobacter pylori* in gastric cancer [15]. *Fusobacterium nucleatum* , a causative agent of periodontal disease, is reportedly present in the stool of patients with colorectal cancer [4]. However, despite the growing body of evidence, a critical gap persists in understanding how specific shifts in oral bacterial composition correlate with clinical and histopathological features of OSCC [16]. Furthermore, limited studies have focused on using 16S rRNA gene sequencing of unstimulated saliva to comprehensively evaluate the oral microbiome in patients with untreated OSCC [17].

Although we previously evaluated the relationship between pathogenic microorganisms in patients with oral cancer and oral potentially malignant disorders (OPMDs) [18, 19, 20, 21], no study has demonstrated a relationship between differences in the bacterial flora and OSCC, with a focus on clinical or histopathological characteristics. Therefore, this study aimed to investigate the relationship between oral microbiota diversity and OSCC using a well‐characterized clinical cohort. Specifically, we sought to clarify how microbiome profiles vary according to tumor stage, nodal involvement, and histopathological grade by employing high‐throughput 16S rRNA sequencing and comprehensive clinical data, thereby providing new insights into the microbiological features of OSCC oncogenesis.

## 2 Materials and Methods

### 2.1 Patients

The study protocol was approved by the Tohoku University Ethics Committee (from the Ethics Committee of the Tohoku University Graduate School of Dentistry; No. 25091, April 27, 2022). Informed consent was obtained from all participants through a standardized process, including oral and written explanations, provision of time to consider participation, opportunities to ask questions, and signing of a written consent form, in accordance with institutional ethical guidelines. The study was conducted in accordance with the principles of the Declaration of Helsinki.

The study population comprised 64 patients and 50 healthy controls who visited the Division of Oral and Maxillofacial Surgery, Department of Tohoku University Hospital, Miyagi, Japan. The 64 patients with OSCCs underwent pathological diagnosis. All patients were referred by their general dental practitioners with a chief complaint of white spots, erythema, pain, and discomfort in the oral cavity. None of the patients with OSCC had received chemotherapy or radiotherapy before surgery. Patients with cancer of the lip, tonsils, larynx, or pharynx, those with tumors of origin other than squamous cells, those with recent antibiotic or topical steroid use, and those unable to provide informed consent were excluded. Healthy controls were included if they had no prior treatment or oral lesions and had no evidence of tumor‐related diseases, oral mucosal diseases, immune diseases, nutritional disorders, or dental infections. Although some healthy individuals had histories of common conditions, such as diabetes mellitus, dyslipidemia, and hypertension, they were included to reflect the diversity of the general population and enhance the external validity of the study.

TNM classification [22], degree of differentiation, stromal lymphocytic reaction, mode of invasion, and invasion depth were evaluated as clinical and histopathological factors for oral cancer. Background characteristics attributable to the oral microflora were investigated at the time of saliva collection, including age, sex, smoking, alcohol consumption, history of alcohol consumption, medical history (diabetes, hyperlipidemia, and hypertension), number of existing teeth, and periodontally affected teeth with a pocket probing depth ≥ 4 mm. The depth of the periodontal pocket probe, which indicates the severity of periodontal disease, was measured on the inner and outer sides of the front and back six teeth. P_Per (%) was defined as the percentage of remaining teeth with a probing depth ≥ 4 mm, calculated by dividing the number of such teeth by the total number of remaining teeth and multiplying by 100, in accordance with the 2017 World Workshop classification [23]. Third molars (wisdom teeth) were excluded for this assessment, and the probing depth was recorded at the deepest site per tooth. The presence of periodontal pockets ≥ 4 mm was used as an indicator of moderate or more severe periodontitis, based on the 2017 World Workshop classification by the American Academy of Periodontology and the European Federation of Periodontology, which defines moderate periodontitis as clinical attachment loss of ≥ 3 mm and/or probing depth ≥ 4–5 mm [23]. Smoking status was categorized as “current smoker” if the participant had smoked within the past 30 days, and as “non‐smoker” if the participant had quit smoking at least 6 months prior to enrollment or had smoked fewer than 100 cigarettes in their lifetime [24]. Alcohol consumption was categorized as “current drinker” if the participant had consumed alcohol within the past 30 days, and as “non‐drinker” if the participant had not consumed alcohol within the past 30 days or had a lifetime alcohol consumption of fewer than 12 occasions [25].

### 2.2 Sample Preparation

All unstimulated whole saliva samples (2 mL) were collected using DNase/RNase‐free, self‐standing 25 mL polypropylene centrifuge tubes (Centrifuge Tubes 25 mL, 2363‐025N, IWAKI, Japan) without any stabilizing agents. Participants were instructed to refrain from eating, drinking, brushing teeth, or using mouthwash for at least 1 h before sampling. Immediately after collection, all saliva specimens were stored at −60°C, delivered to Tohoku University Biobank (Sendai City, Miyagi Prefecture), and subsequently frozen at −80°C after aliquoting. The saliva samples were then delivered to Tohoku University Tohoku Medical Megabank Organization Research Institute (Sendai City, Miyagi Prefecture) and stored frozen at −80°C until analysis.

### 2.3 Genomic DNA Extraction

The composition and diversity of the oral microbiome were assessed by high‐throughput sequencing of the 16S rRNA gene amplicons using the Illumina MiSeq platform (Illumina Inc., San Diego, CA, USA). DNA was extracted from saliva samples using a DNeasy Powersoil Pro kit (QIAGEN Inc., Hilden, Germany) according to the manufacturer's protocol. Sequencing libraries were prepared using a two‐step PCR method targeting the V3–V4 hypervariable region of the 16S rRNA gene. PCR amplification was performed using Takara ExTaq (TaKaRa Bio Inc., Shiga, Japan). PCR was performed using the following gene‐specific primers: forward primer, 5′‐ACACTCTTTCCCTACACGACGCTCTTCCGATCTNNNNNCCTACGGG‐NGGCWGCAG‐3′; and reverse primer, 5′‐GTGACTGGAGTTCAGACGTGTGCTCTTCCGATCTNNNNNGACTACHVGGGTATCTAATCC‐3′. The specifics of the PCR process are outlined below: the temperature was set to 94°C for 3 min, followed by 30 cycles of 94°C for 30 s, 55°C for 30 s, and 72°C for 30 s. This was followed by an extension period of 72°C for 5 min and a final hold at 4°C. Subsequently, 3 μL of the 20 μL amplification product and 2% agarose gel were used to ascertain the presence of PCR bands, which were identified at 540 bp.

Thereafter, a second PCR was performed using distinct indexing primers that integrated the Illumina sequencing adapters and dual barcodes into the amplicon. The pooled library was quantified using a Qubit 2.0 Fluorometer and a dsDNA HS Assay Kit (Life Technologies, Carlsbad, CA, USA) before being diluted to a final concentration of 12 pM with 50% PhiX. Sequencing was conducted using a MiSeq Reagent Kit v3 (Illumina Inc.) with a 260 bp single‐end sequencing protocol in accordance with the manufacturer's instructions. In total, 2.7 million single‐end reads were generated. The mean read pair count for the samples was 24 065, with a maximum of 82 113 read pairs.

### 2.4 Amplicon Sequence Variants (ASVs)

Sequence data for the 16S rRNA gene amplicons were analyzed using the Quantitative Insights Into Microbial Ecology 2 (QIIME2) platform, version 2024.2. The initial 20 bases of both sequences were excised to remove primer sequences for all paired reads. Additionally, bases subsequent to position 200 were truncated to remove low‐quality sequence data, and potential amplicon sequencing errors were corrected using DADA2 to generate an ASV dataset. The ASV results were aligned using the MAFFT software (version 7.526) and subsequently employed to construct a phylogenetic tree using FastTree2. Alpha‐ and beta‐diversity metrics were estimated from a subsampled ASV dataset, utilizing 1000 sequences per sample. Each ASV was identified using a Naïve Bayes classifier, which was trained on the 16S rRNA gene sequences from the Greengenes2 dataset. Fifteen ASVs were assigned to the phylum level, 19 to the class level, 191 to the genus level, and 386 to the species level.

**FIGURE 1:**
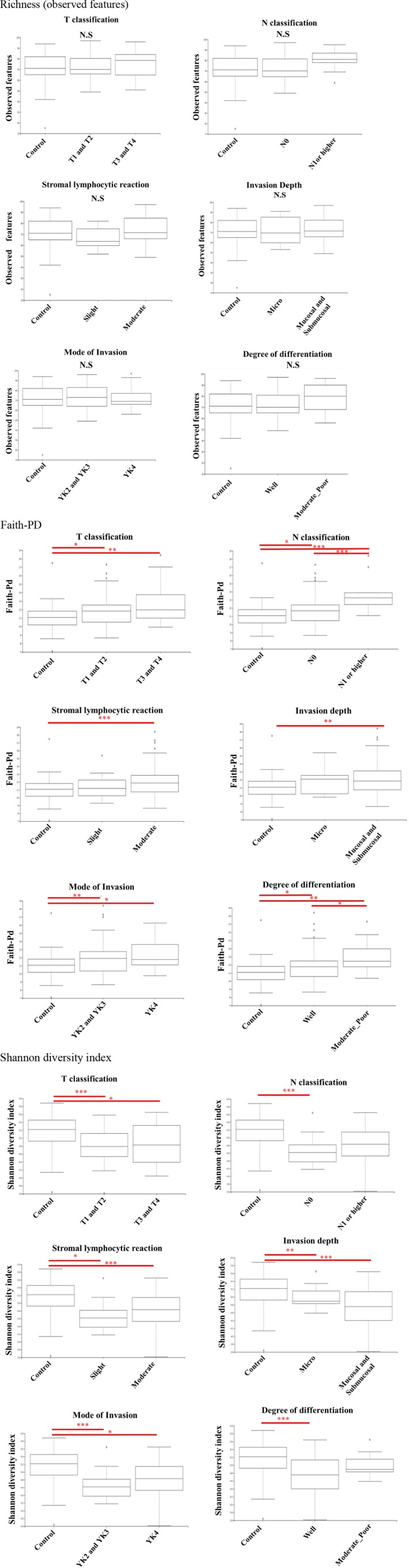
Alpha diversity indices (richness, Shannon index, Faith's PD) of salivary microbiota in OSCC patients (*n* = 64) and healthy controls (*n* = 50). The Shannon diversity index is a widely used alpha diversity metric and a measure of information, which can be thought of as the uncertainty in whether two random individuals in a sample/community are similar. If all taxa have the same abundance, then evenness is high, if one or few taxa dominate others, then evenness is low. Faith's phylogenetic diversity (Faith's PD) is an alpha diversity metric which incorporates the phylogenetic tree of the taxa. Faith's PD is the sum of all the branch lengths that are connecting all taxa observed in the sample. Box plots of observed features, Shannon entropy and Faith phylogenetic diversity in the saliva of each group categorized by pathological diagnosis are shown. Observed feature indicates the index of all amounts of Operational Taxonomic Units (OTUs) detected. Shannon entropy indicate the evenness of species in each community. The faith phylogenetic diversity indicates the diversity index calculated based on a phylogenetic tree. *(*p* < 0.05) and the **(*p* < 0.01) indicate significant differences calculated using Steel–Dwass method.

**FIGURE 2:**
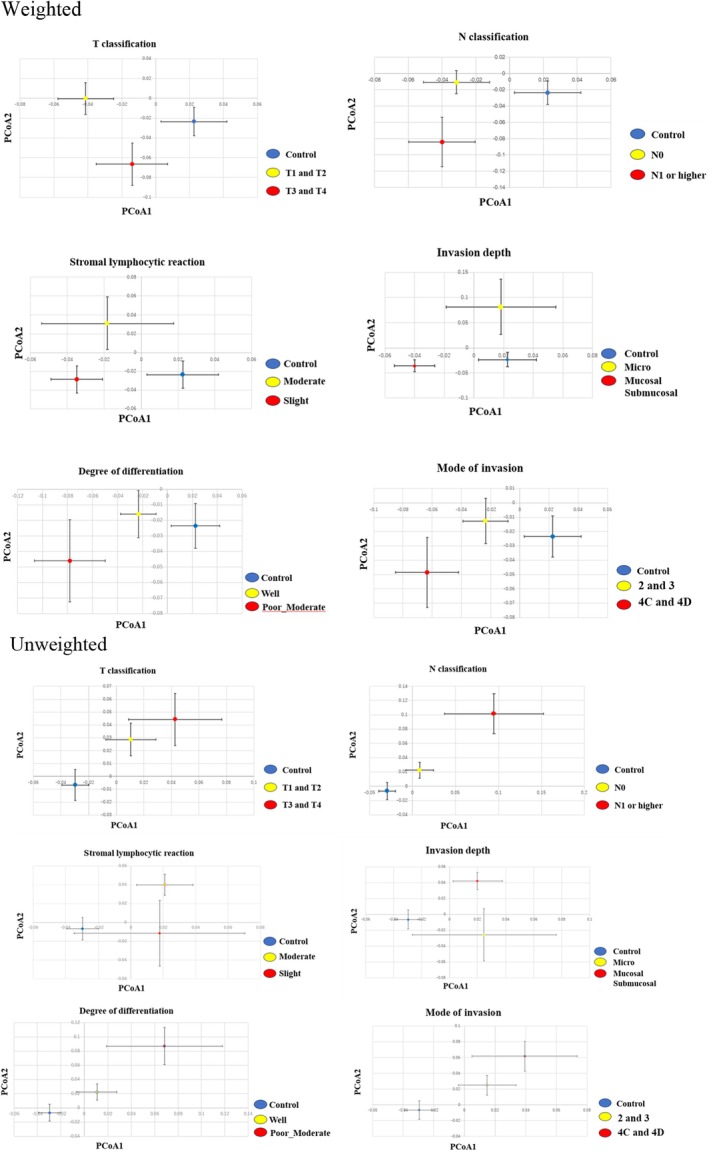
Principal coordinates analysis (PCoA) of salivary microbiota based on weighted and unweighted UniFrac distances in OSCC patients (*n* = 64) and healthy controls (*n* = 50). Each point in the PCoA plot represents a sample, with the data being projected onto a two‐dimensional space by PCoA1 and PCoA2. Clusters in different colors represent different groups, making it possible to visualize similarities between samples and the separation between groups. Weighted Unifrac considers the lead count (evaluated with lead count weight)m while Unweighted Unifrac does not consider the lead count. Each plot in the PCoA, based on weighted and unweighted unifrac distances, shows the mean of value of the principal coordinates in each category categorized by pathological diagnosis, while the bars indicate standard divisions. Weighted PCoA considers the read count whereas unweighted PCoA does not consider read count.

Alpha‐diversity indices, including the observed ASVs, richness, Shannon's index, and Faith's phylogenetic distance (Faith‐PD), were calculated (Figure 1). The observed richness is defined as the number of different taxa in a community and is the simplest alpha diversity metric. The Shannon diversity index is a widely used alpha diversity metric as a measure of information and can be considered the uncertainty of whether two random individuals in a sample/community are similar. If all taxa have the same abundance, then evenness is high; if one or few taxa dominate, the evenness is low. Faith‐PD incorporates the phylogenetic tree of the taxa and is the sum of all branch lengths that connect all taxa observed in the sample. Beta‐diversity was evaluated using UniFrac principal coordinates‐based Principal Coordinates Analysis (PCoA) (Figure 2). Data were projected onto a two‐dimensional space using PCoA1 and PCoA2. Weighted and unweighted UniFrac distances were calculated to assess beta‐diversity. The read count (i.e., the abundance of each taxon) was incorporated as a weighting factor in the weighted UniFrac and PCoA, reflecting the presence and relative abundance of taxa. In contrast, unweighted UniFrac and PCoA considered only the presence or absence of taxa without accounting for read count. Clusters of different colors represent separate groups, allowing for the visualization of similarities between samples and differences between groups. The mean PC values for each group along the *x*‐ and *y*‐axes are indicated by circular shapes, and the 95% confidence intervals (CI) for the means are indicated by whiskers. The microbiome data are represented by the relative abundance at each taxonomic level, and between groups comparisons were performed for all observed bacteria.

### 2.5 Statistical Analysis

All statistical analyses were conducted using JMP 17.0. The Steel–Dwass test was employed as a nonparametric test to compare three or more groups. The differential abundance of genera among groups was identified using the Analysis of Compositions of Microbiomes with Bias Correction (ANCOM‐BC) method. *p* < 0.05 was considered statistically significant.

## 3 Results

### 3.1 Patient Characteristics

The clinical characteristics of the healthy controls and patients with OSCC are shown in Table S1 and Table S2. TNM disease stages were obtained from medical records and classified according to the Union for International Cancer Control system. No patient in the control group was diagnosed with SCC at any site prior to surgery. During the follow‐up period (12–48 months), no patient in the control group experienced OSCC. The 64 patients with OSCC were pathologically classified according to the World Health Organization classification of tumors of the oral cavity and mobile tongue [26].

The background characteristics attributable to the oral microflora showed a bias in each group with regard to smoking or alcohol consumption (chi‐square, *p* < 0.001); however, no other significant differences were observed. All participants were followed up through medical records for a period of 12–48 months to confirm the absence of OSCC in the control group. No follow‐up microbiome sampling or repeat measurements were performed during this period. All microbiome analyses were based solely on baseline unstimulated saliva samples collected at enrollment.

### 3.2 Alpha Diversity

Although no significant differences were observed in richness (number of characteristics observed), OSCC cases were higher in patients with larger T3/T4 tumor diameters, lymph node metastases, and histopathologically poorly differentiated cases than in healthy controls. The Shannon diversity index was significantly higher in the control group than that in the OSCC group; however, no significant differences were observed among OSCC cases in the T and N classification, degree of differentiation, and stromal lymphocytic reaction. Faith‐PD was higher in the OSCC group than that in the control group. Furthermore, among patients with OSCC, those with a poor histopathological grade of differentiation and those with clinical lymphatic metastasis (N1 or higher) had significantly higher Faith‐PD values. No significant differences were observed in Faith‐PD values among OSCC cases according to T classification, stromal lymphocytic reaction, mode of invasion, or invasion depth; however, Faith‐PD was higher in cases with a higher clinicopathologic grade of malignancy.

### 3.3 Beta Diversity

OSCC cases were separated from the control group in the weighted and unweighted PCoAs, forming clusters. In the weighted PCoA, patients with OSCC with histopathologically deeper submucosal and mucosal invasion, clinically larger T3/T4 tumor diameter, and lymph node metastasis (N1 or greater) formed separate independent clusters from those with smaller tumor diameters and without lymph node metastasis (N0). In the unweighted PCoA, patients with OSCC with clinical lymph node metastasis (N1 or greater) formed a significantly separate cluster from patients without lymph node metastasis (N0).

### 3.4 Class‐Level Microbiome

**FIGURE 3:**
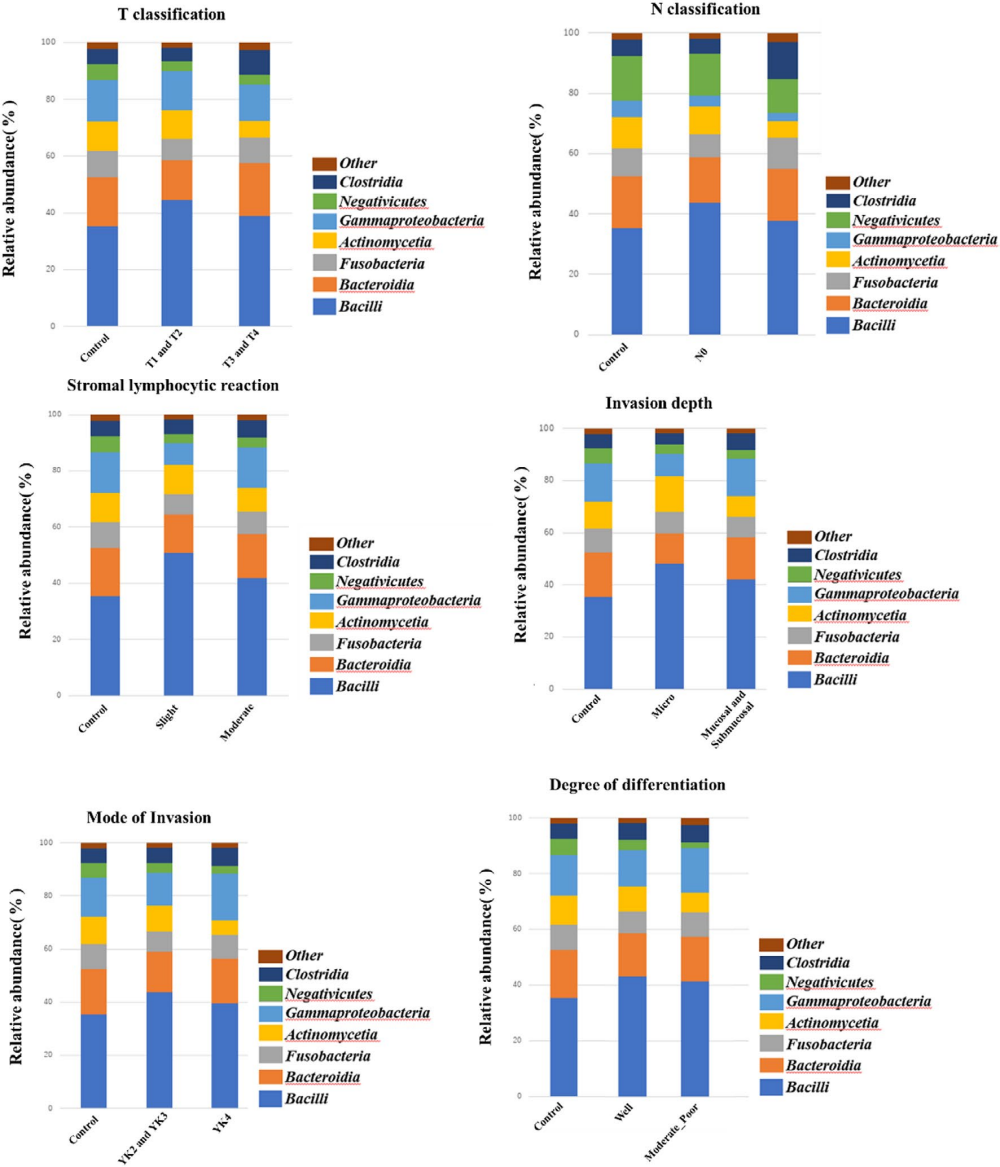
Relative abundance of salivary microbiota at the class level according to clinical stage and histopathological grade in OSCC patients (*n* = 64) and healthy controls (*n* = 50). Relative abundances of different bacterial taxa in each malignancy category are shown. Each color corresponds to a different bacterial group, and the height of each colored area represents the percentage of the bacterial group in the sample from each site. Steel–Dwass tests were used to analyze each of the grading categories to identify the significantly more abundant bacterial taxa: *Bacilli* increased in the OSCC group compared to the control group regardless of the grading category, while *Negativicutes* and Actinomyces decreased in the OSCC group compared to the control group. *Clostridia* levels also increased in the OSCC group. In addition, the prevalence of *Actinomycetia* was significantly lower in patients with OSCC with larger T3 and T4 tumors than in those with smaller T1 and T2 tumors. The prevalence of *Clostridia* was significantly increased in patients with OSCC with lymph node metastasis (N1, N2, or higher) than in controls and patients with OSCC without lymph node metastasis. The figure shows the relative abundance of microbial taxa at class level. Stacked bar plots and the relative abundance of major bacterial species (more than 1%) at class level in each diagnosed category are shown. Less than 1% of the species were classified as Others.

Stacked bar graphs focusing on the microbiome (class) by clinical and histopathological grade are shown in Figure 3. Steel–Dwass tests were performed for each of the grading categories to identify the significantly more abundant bacterial taxa. The OSCC group had more *Bacilli*, regardless of the grading category, and fewer *Negativicutes* and *Actinomyces* compared to the control group. *Clostridia* levels were also increased in the OSCC group, specifically in patients with OSCC with lymph node metastasis (N1, N2, or higher) than those in controls and patients with OSCC without lymph node metastasis. Conversely, the prevalence of *Actinomycetia* was significantly lower in patients with OSCC with larger T3 and T4 tumors than in those with smaller T1 and T2 tumors.

### 3.5 Heat Map

**FIGURE 4:**
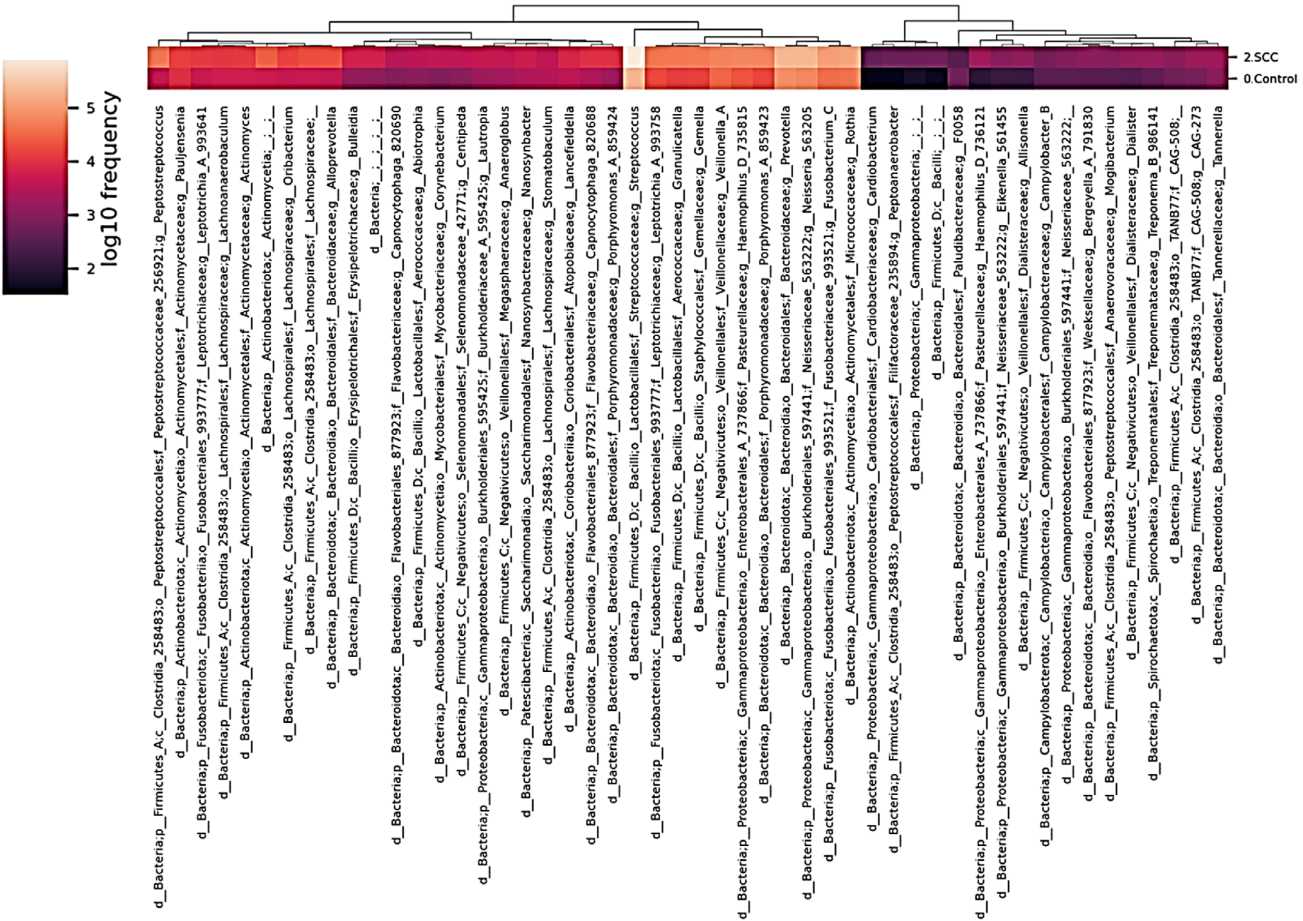
Heatmap of salivary microbiota composition at the genus level comparing OSCC patients (*n* = 64) and healthy controls (*n* = 50). The darker the orange color, the more abundant the species, and the darker the purple color, the less abundant the species. A heatmap of the salivary microbiome composition comparing the control and OSCC groups is shown. This optimized heatmap illustrates microbial features, selected and ranked using machine learning, to predict the characteristics of the salivary microbiomes of the two groups. Each column represents a microbial feature at genus level, while rows correspond to each group. The color gradient corresponds to the log_10_ frequency of microbial features, with darker colors representing lower abundance. Sample clustering is shown in the dendrogram at the top.

A heat map was generated to depict the abundance of each bacterial species at the genus level (Figure 4). The darker the orange color, the more abundant the species, and the darker the purple color, the less abundant the species. The rows show the major bacterial species, whereas the columns contain the four groups. When a tree was drawn on top of the heat map, species with biological similarity generally clustered according to each group. However, differences in the distribution of the overall bacterial flora between the groups were not significant.

### 3.6 ANCOM‐BC

**FIGURE 5:**
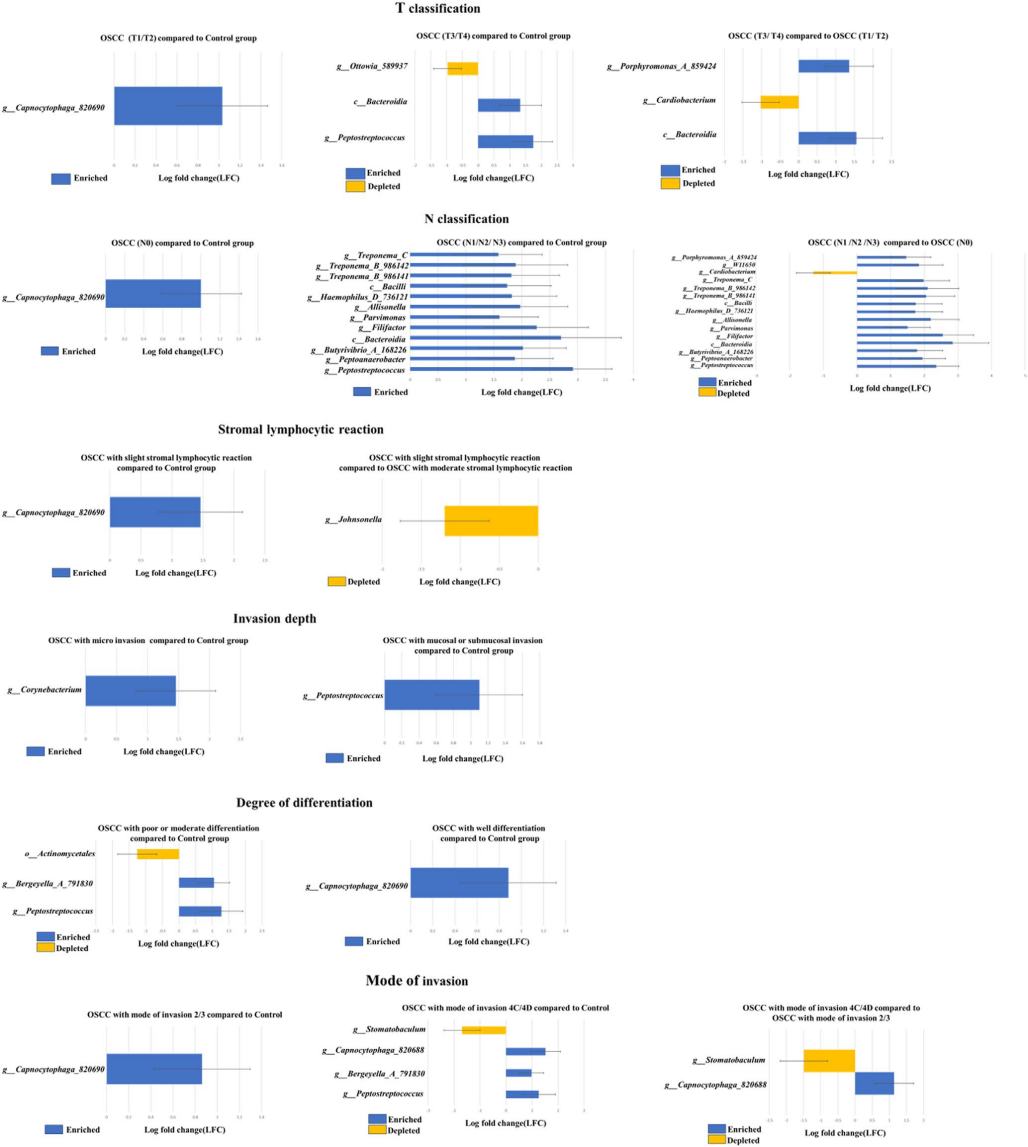
Differentially abundant salivary microbial taxa identified by ANCOM‐BC according to clinical stage and histopathological grade in OSCC patients (*n* = 64) and healthy controls (*n* = 50). Several bacterial species showed a significant increase or decrease with changes in histopathological grade and TNM classification. Analysis of Composition of Microbiomes with Bias Correction (ANCOM‐BC) in salivary microbiome between each clinical characteristics and pathological grade is shown. The results of effect size (log fold change) and 95% confidence intervals (CI) using ANCOM‐BC package are shown. Positively and negatively changed pathways are indicated in pale bule and pale yellow bars, respectively.

We examined the differentially abundant taxa among patients with OSCC grouped by clinical and histopathologic grade compared to the control group (Figure 5). ANCOM‐BC showed the differential abundance of bacteria in the samples between each clinical characteristic and pathological grade (*p* < 0.05). Additionally, ANCOM‐BC was used to assess differences between samples from each group and identify bacteria that were significantly more or less abundant.

## 4 Discussion

Bacteria may be involved in the transformation of “normal cells” into “cancer cells” in other cancers, just as *H. pylori* is involved in gastric cancer development [27]. Therefore, we aimed to evaluate the relationship between oral microbiota and the development of oral cancer. We employed alpha diversity indices because they can be broadly categorized into the number of species (richness) and the evenness of their distribution (evenness). Richness captures the total number of species present, Faith‐PD incorporates phylogenetic diversity, and the Shannon index accounts for the richness and evenness of species distribution. We found significant differences in Faith‐PD between healthy controls and patients with OSCC before treatment, indicating that bacterial flora diversity is greater in patients with OSCC than in healthy controls. Additionally, an increasing trend in richness was observed, suggesting a rise in the number of bacterial species as OSCC develops. Among patients with OSCC, Faith‐PD values were significantly higher in those with low histopathological differentiation and clinical lymph node metastases (N1 or higher) than in their counterparts. Additionally, a characteristic increase in diversity was observed among patients with OSCC in those with a higher clinicopathological grade compared to those with a lower clinicopathological grade. PCoA also separated patients with OSCC from controls with healthy oral mucosa, forming a special cluster. Moreover, among patients with OSCC, those with histopathological submucosal invasion, clinically large T3/T4 tumor diameter, and lymph node metastasis (N1 or greater) were independently isolated. Thus, we believe that our results identify the unique bacterial flora transition specific to oral cancer that first increases in diversity at tumor onset and then matures into characteristic clusters as it progresses.

Notably, no differences were observed in the bacterial microbiota between patients with OSCC and the control group in terms of age and sex. However, according to Han et al. [13] while normal saliva microbiomes exhibit clear age‐ and sex‐related characteristics, patients with OSCC show disruption of these age‐ and sex‐related microbiome signatures. This suggests that OSCC may alter the complex interactions between the host and microorganisms, thereby impairing the normal aging patterns of the microbiome.

Additionally, the specimen type can significantly influence microbiome profiles. While we used saliva samples, Wang et al. [14] analyzed tumor‐adjacent mucosal tissues in OSCC and reported microbial patterns distinct from those found in saliva. These differences may reflect local immune activity and bacterial adhesion at tissue sites. In contrast, salivary samples reflect a composite of microbes from various oral niches. Thus, our findings highlight the need to interpret salivary microbiome data within the context of its sample type and suggest that specimen selection is critical for understanding site‐specific microbial alterations in OSCC.

Cai et al. showed that the composition of oral flora in patients with recurrent aphthous stomatitis (RAS) changed at various stages of the disease course but with reduced diversity compared to healthy individuals [27]. Similarly, our analysis revealed that bacterial diversity increased in patients with deep submucosal invasion or ulceration of large‐diameter oral cancers, such as T3 and T4 tumors, compared to their counterparts. This suggests that the diversity and composition of the bacterial flora differ according to distinct disease processes compared to that in ulceration caused by stomatitis, wherein the diversity of the flora tends to decrease.

The taxonomic rank of biological classification in our study showed that *Negativicutes* and *Actinomyces* were lower and *Bacilli* were higher in the OSCC group compared to the control group, regardless of the grading category. Additionally, patients with OSCC with lymph node metastasis (N1, N2, or higher) exhibited a higher prevalence of *Clostridia* compared to the control group and patients with OSCC without lymph node metastasis. Conversely, the prevalence of *Actinomycetia* was significantly lower in patients with OSCC with larger T3 and T4 tumors than in those with smaller T1 and T2 tumors. *Actinobacteria*, especially genera *Rothia* and *Actinomyces*, were significantly underrepresented in cases with higher stages and pathological grades than in those with lower stages and pathological grades, whereas phyla *Spirochaetia* and *Proteobacteria*, especially genus *Treponema*, were significantly overrepresented in advanced cancer compared to early stage cancer. At the species level, *Prevotella histicola* , *Treponema denticola* , *Actinomyces graevenitzii* , *Treponema lecithinolyticum* , and *Peptostreptococcus stomatisna* were significantly more common in advanced cancer than in early stage cancer.

Thus, our results indicate that the transition of oral microflora, such as *Actinomycetia* and *Spirochaetia*, may be involved in the development and progression of oral cancer. The *Spirochaetia* species may directly promote cancer cells and invasive metastasis [28], since they, particularly *T. denticola* (Td), can alter the activity of immunomodulatory proteins such as matrix metalloproteinases and their tissue inhibitors that play important roles in the tumor microenvironment [29]. Moreover, some oral bacteria have mechanisms to evade host immune surveillance and promote tumor growth [30]. Bacterial oral microbiome imbalances also develop various mechanisms that promote their ability to establish themselves in the gastrointestinal tract, including strong adhesion, immune evasion, disruption of epithelial integrity, and intracellular survival [31]. For instance, Gallimidi et al. [30] suggested that oral bacteria directly influence cell behavior by regulating cell proliferation, apoptosis, and cell invasion. In a study using 16S ribosomal RNA high‐throughput sequencing to detect differences in the oral mucosal flora of patients with RAS and healthy participants, Cai et al. showed that the number of Gram‐negative anaerobic bacteria *Prevotella* belonging to the phylum *Bacteroides* was significantly increased at the ulcer site of RAS compared to healthy oral mucosa [27]. In this regard, we found that oral cancer differs from stomatitis with ulceration, which has decreased bacterial diversity, in that oral cancer has expanded bacterial flora diversity compared to healthy mucosa. Furthermore, the composition of the bacterial flora specific to oral cancer differs from that of stomatitis, suggesting that the bacterial flora changes according to the cause of the ulcer in the oral mucosa and that cancer cells may be involved in the transition of the flora.

Notable strengths of this study include the fact that all patients were examined and analyzed under the same pretreatment conditions, no antibiotics or antifungals were used as the gold standard for comparison, and the patient sample was homogeneous. Nonetheless, this study has some limitations. For instance, patients with stomatitis and OPMD were not analyzed; only healthy participants and patients with early and advanced oral cancer were compared. Additionally, we were unable to analyze the changes in bacterial flora by each oral site in detail because the number of cases of carcinoma of the floor of the mouth and buccal mucosa in this study was somewhat smaller than that of tongue cancer. Moreover, since this was an observational study, we could not establish causal relationships between changes in the microbiota and oral cancer development or progression. Therefore, experimental and interventional studies are required to determine causality. Furthermore, although this study identifies associations between microbiota changes and oral cancer, it does not delve deeply into the molecular mechanisms underlying these changes. Additionally, we only analyzed the bacterial component of the oral microbiome. Other microbiome constituents, such as fungi and viruses, were not assessed, which may limit the comprehensiveness of the microbial community analysis. Consequently, further research is required to elucidate these mechanisms. Another key limitation of this study is the relatively small number of samples in some advanced cancer subgroups, particularly in the T4 and N2 categories, which may limit the statistical power to detect microbiome differences related to disease extent and may introduce variability in subgroup‐specific findings. Moreover, as this was a cross‐sectional study, our findings cannot determine the directionality of the association between microbiota composition and OSCC. Therefore, longitudinal studies are required to clarify temporal relationships since we cannot infer whether observed microbiota differences are a cause or consequence of OSCC. Furthermore, the inclusion of individuals with chronic diseases such as diabetes mellitus and hypertension may have introduced confounding effects on the oral microbiome. Smoking and alcohol use were also not fully adjusted for and may have partially affected the observed associations. Therefore, future studies should control for such confounders through stricter inclusion criteria or statistical adjustment. Finally, our study was a single‐center study of a small number of patients which was inadequate to conclude an association between a very limited bacterial flora and oral cancer. Consequently, future large‐scale prospective studies are required to address these limitations.

I have revised this sentence to maintain the formal and native tone of the text.

In conclusion, differences in bacterial flora observed in this study are associated with the presence and clinical features of OSCC. However, future large‐scale, multi‐regional cohort studies are required to validate these findings and assess the regional and demographic variations in oral microbiota associated with oral cancer.

## Author Contributions

S.F., K.Y., and T.S. contributed to design, data acquisition, analysis, interpretation, drafted, and critically revised the manuscript. A.K. and T.T. contributed to conception, design, data acquisition, analysis, interpretation, drafted, and critically revised the manuscript. T.S., A.I., Y.H., T.K., T.K., and M.M. contributed to analysis and critically revised the manuscript. M.I., R.S., and T.T. contributed to design, data acquisition, and drafted the manuscript. All authors gave their final approval and agreed to be accountable for all aspects of the work.

## Funding

The authors have nothing to report.

## Conflicts of Interest

The authors declare no conflicts of interest.

## Supporting information


**Table S1:** Clinico‐pathological features in 64 OSCC cases.
**Table S2:** Clinical characteristics of the OSCC and control groups.

## Data Availability

The data that support the findings of this study are available on request from the corresponding author. The data are not publicly available due to privacy or ethical restrictions.
