# Supplementary material for: Differences in the Oral Microbiome Between Patients With and Without Oral Squamous Cell Carcinoma
Source: J Oral Pathol Med. 2025 Dec 5;55(3):368–80. doi: 10.1111/jop.70099 (PMC12963696; doi:10.1111/jop.70099)
Supplement: Supplementary file 1 — Table S1: Clinico‐pathological features in 64 OSCC cases. Table S2: Clinical characteristics of the OSCC and control groups. [file JOP-55-368-s001.docx]

**Title**

Oral microbiome changes in oral squamous cell carcinoma oncogenesis

**Author names**

Satoshi Fukase, Atsumu Kouketsu, Toru Tamahara, Tatsuru Saito, Akiko Ito, Yutaro Higashi, Tomonari Kajita, Tsuyoshi Kurobane, Masaaki Miyakoshi, Masahiro Iikubo, Ritsuko Shimizu, Tetsu Takahashi, Kensuke Yamauchi, Tsuyoshi Sugiura

Supplementary Table 1 Clinico-pathological features in 64 OSCC cases.

|  |  |  | Number of cases | |
| --- | --- | --- | --- | --- |
| All cases |  |  | 64 |  |
|  |  |  |  |  |
| T classification | 1 |  | 20 |  |
|  | 2 |  | 24 |  |
|  | 3 |  | 12 |  |
|  | 4 |  | 8 |  |
|  |  |  |  |  |
| N classification | 0 |  | 55 |  |
|  | 1 |  | 5 |  |
|  | 2 |  | 3 |  |
|  | 3 |  | 1 |  |
|  |  |  |  |  |
| M classification | 0 |  | 64 |  |
|  | 1 |  | 0 |  |
|  |  |  |  |  |
| Degree of Differentiation | Poor |  | 3 |  |
|  | Moderate |  | 8 |  |
|  | Well |  | 53 |  |
|  |  |  |  |  |
| Stromal lymphocytic reaction | Slight |  | 8 |  |
|  | Moderate |  | 56 |  |
|  |  |  |  |  |
| Mode of invasion | 2 |  | 18 |  |
|  | 3 |  | 31 |  |
|  | 4C |  | 12 |  |
|  | 4D |  | 3 |  |
|  |  |  |  |  |
| Invasion depth | Micro |  | 8 |  |
|  | Mucosal |  | 27 |  |
|  | Submucosal |  | 29 |  |

Supplementary Table 2. Clinical characteristics of the OSCC and control groups

|  |  | Control (N=50) | OSCC (N=64) | | | | | | | | | | | |
| --- | --- | --- | --- | --- | --- | --- | --- | --- | --- | --- | --- | --- | --- | --- |
|  |  |  | T | | N | | Stromal lymphocytic reaction | | Invasion depth | | Mode of invasion | | Degree of differentiation | |
|  |  |  | T1/T2 | T3/T4 | N0 | N1/N2/N3 | slight | moderate | micro | mucosal/submucosal | 2/3 | 4C/4D | Well | Poor/  Moderate |
| **Age** | years | 60.3 ± 12.4 | 62.2 ± 16.5 | 67.8 ± 13 | 64.9 ± 16 | 58.2 ± 12.3 | 69.4 ± 17.6 | 63.2 ± 15.3 | 62.1 ± 18 | 64.2 ± 15.4 | 65 ± 16.4 | 60.4 ± 12.5 | 64.2 ± 16.4 | 62.8 ± 11.5 |
|  |  |  |  |  |  |  |  |  |  |  |  |  |  |  |
| **Sex** | Male | 20 | 20 | 11 | 27 | 4 | 3 | 28 | 5 | 26 | 22 | 9 | 22 | 9 |
|  | Female | 30 | 24 | 9 | 28 | 5 | 5 | 28 | 3 | 30 | 27 | 6 | 31 | 2 |
|  |  |  |  |  |  |  |  |  |  |  |  |  |  |  |
| **Drinker** | Current | 19 | 17 | 11 | 23 | 5 | 4 | 24 | 2 | 26 | 20 | 8 | 19 | 9 |
|  | Never | 31 | 26 | 9 | 31 | 4 | 4 | 31 | 6 | 29 | 29 | 6 | 33 | 2 |
|  | Unknown | 0 | 1 | 0 | 1 | 0 | 0 | 1 | 0 | 1 | 0 | 1 | 1 | 0 |
|  |  |  |  |  |  |  |  |  |  |  |  |  |  |  |
| **Smoker** | Current | 15 | 15 | 12 | 22 | 5 | 3 | 24 | 3 | 24 | 19 | 8 | 20 | 7 |
|  | Never | 35 | 28 | 8 | 32 | 4 | 5 | 31 | 5 | 31 | 30 | 6 | 32 | 4 |
|  | Unknown | 0 | 1 | 0 | 1 | 0 | 0 | 1 | 0 | 1 | 0 | 1 | 1 | 0 |
|  |  |  |  |  |  |  |  |  |  |  |  |  |  |  |
| **Diabetes** | Current | 7 | 8 | 3 | 10 | 1 | 3 | 8 | 2 | 9 | 7 | 4 | 7 | 4 |
|  | Never | 43 | 36 | 17 | 45 | 8 | 5 | 48 | 6 | 47 | 42 | 11 | 46 | 7 |
|  |  |  |  |  |  |  |  |  |  |  |  |  |  |  |
| **Hyperlipidemia** | Current | 12 | 8 | 4 | 11 | 1 | 1 | 11 | 1 | 11 | 9 | 3 | 9 | 3 |
|  | Never | 38 | 36 | 16 | 44 | 8 | 7 | 45 | 7 | 45 | 40 | 12 | 44 | 8 |
|  |  |  |  |  |  |  |  |  |  |  |  |  |  |  |
| **Hypertension** | Current | 22 | 20 | 14 | 29 | 5 | 3 | 31 | 1 | 33 | 25 | 9 | 27 | 7 |
|  | Never | 28 | 24 | 6 | 26 | 4 | 5 | 25 | 7 | 23 | 24 | 6 | 26 | 4 |
|  |  |  |  |  |  |  |  |  |  |  |  |  |  |  |
| **Number of existing teeth** |  | 23.8 ± 5.5 | 21.4 ± 8 | 20.8 ± 8.5 | 21.4 ± 8 | 20.1 ± 9.1 | 20.1 ± 8.1 | 21.4 ± 8.2 | 26.3 ± 3.8 | 20.5 ± 8.3 | 21.6 ± 7.8 | 20.2 ± 9 | 21.2 ± 8.1 | 21.5 ± 8.2 |
|  |  |  |  |  |  |  |  |  |  |  |  |  |  |  |
| **P_Per (%)** |  | 21.7 ± 16.8 | 30.2 ± 26.7 | 37.9 ± 28.2 | 32 ± 27.4 | 36.2 ± 27.1 | 39.1 ± 31.7 | 31.8 ± 26.8 | 16.4 ± 8.9 | 35.1 ± 28.2 | 31.7 ± 27.9 | 35.4 ± 25.6 | 29.9 ± 25.3 | 46 ± 33.3 |
|  |  |  |  |  |  |  |  |  |  |  |  |  |  |  |
